# Anatomical Validation and Technical Feasibility of Biportal Endoscopic Spinal Surgery Including Technical Notes in a Cadaveric Canine Thoracic Intervertebral Disc Disease Model

**DOI:** 10.3390/ani16030435

**Published:** 2026-01-30

**Authors:** Sung-Ho Lee, Ji-Hyun Park, Da-Eun Kim, Gunha Hwang, Chang-Hwan Moon, Dongbin Lee

**Affiliations:** 1 Department of Veterinary Surgery, College of Veterinary Medicine, Gyeongsang National University, 501, Jinjudae-ro, Jinju 52828, Republic of Korea; ho911001@naver.com (S.-H.L.); 24qkr@naver.com (J.-H.P.); ekdmsaa_a@naver.com (D.-E.K.); ch.moon@gnu.ac.kr (C.-H.M.); 2 Department of Veterinary Medical Imaging, College of Veterinary Medicine, Gyeongsang National University, 501, Jinjudae-ro, Jinju 52828, Republic of Korea; hgh3634@naver.com

**Keywords:** biportal endoscopic spinal surgery, veterinary spinal surgery, canine thoracic spine, minimally invasive spinal surgery, mini-hemilaminectomy, intervertebral disc disease, dog

## Abstract

Biportal endoscopic spinal surgery (BESS) is a minimally invasive technique that is widely used in human spinal procedures; however, its standardized methodology and anatomical adaptation for veterinary applications have not yet been established. This study aimed to develop a reproducible experimental framework for performing BESS in dogs and evaluate its technical feasibility. A thoracic intervertebral disc disease model was created by injecting a fluorescently dyed artificial disc material containing methylene blue into the T12–13 intervertebral space of 13 medium-sized canine cadavers. Portal locations were determined using a computed tomography-based measurement method, and instruments specifically designed for BESS were used to perform mini-hemilaminectomies of the accessory process. The artificial disc material was successfully removed in all cases with clear visualization of the spinal cord and nerve roots. The mean portal insertion angle and distance were 31.00 ± 2.79° and 32.95 ± 3.05 mm, respectively, and the average residual material volume was 6.89% ± 1.66% of the initially inserted volume. Surgical time significantly decreased as the surgeon’s experience increased. These results demonstrate the successful methodological standardization of BESS tailored to canine thoracic anatomy and provide foundational data supporting its potential as a minimally invasive spinal surgery technique for future clinical veterinary applications.

## Simple Summary

Thoracic intervertebral disc disease commonly affects small to medium-sized dogs, particularly the T12–13 disc space. Traditional spinal surgery requires wide incisions and extensive osteotomy, which can cause greater soft tissue damage and require longer recovery. In human medicine, a less invasive method, known as biportal endoscopic spinal surgery, is commonly used. However, despite the growing interest in its veterinary application, standardized methodology and anatomical guidelines have not yet been established. This study aimed to establish a reproducible and standardized experimental methodology for performing biportal endoscopic spinal surgery in dogs, using a thoracic intervertebral disc disease model. Fluorescently dyed artificial disc material was injected into the thoracic spine to simulate disc disease, and a computed-tomography-based measurement method was used to determine precise portal locations. The material was successfully removed from all the dogs, and the spinal cord and nerve roots were clearly visualized. These findings provide a foundation for the methodological standardization of biportal endoscopic spinal surgery in veterinary medicine. They may also facilitate its future clinical application as a minimally invasive spinal surgery technique.

## 1. Introduction

Intervertebral disc disease (IVDD) in dogs is a common ailment that compresses the spinal cord [1,2,3,4]. It typically occurs in chondrodystrophic and non-chondrodystrophic breeds owing to genetic or environmental factors, leading to disc protrusion or extrusion [5,6]. This condition predominantly manifests as thoracolumbar disc lesions in medium and small breeds, with an incidence as high as 87%. It predominantly affects dogs weighing approximately 10 kg, particularly in the T12–13 spinal region [2,7,8].

When medical treatment or management is ineffective, traditional surgical methods involve extensive incisions in the skin, muscles, ligaments, and other related soft tissues to reach the affected disc. To minimize the extensive damage to soft tissues in spine surgery, minimally invasive spine surgery (MISS) has evolved significantly in both human and veterinary medicine [9,10,11,12,13,14,15,16,17,18,19,20]. These include methods such as microscopic and endoscopic spinal surgeries. Two main types of endoscopic spinal surgery exist: uniportal endoscopic spinal surgery (UESS), which is performed through a single hole, and biportal endoscopic spinal surgery (BESS), which uses two holes [21,22].

The MISS was first attempted in 1977 by Casper and Yasargil, and the first fully functional endoscopic system was developed and used by Yeung in the late 1990s [23,24,25]. In 1996, De Antoni introduced the first surgery involving the insertion of two portals [26]. This was followed by the introduction of the unilateral biportal endoscopic technique by Soliman in 2013, which is similar to the current BESS technique [27]. Since then, research in this field has rapidly expanded. Recent studies have shown that MISS lowers morbidity, mortality, and disability compared with traditional spinal surgery [28,29]. It also offers the advantages of faster recovery and shorter hospital stays [28,30]. Given these benefits, MISS has been the subject of extensive research in human medicine, which has led to high-quality outcomes. It has also gained popularity in veterinary medicine [17,18,31].

To date, veterinary research on minimally invasive spinal surgery (MISS) has focused primarily on UESS. More recently, interest in BESS has begun to emerge in veterinary medicine. However, despite a few preliminary studies in dogs [9,11,32], standardized surgical methodology and anatomical guidelines for veterinary application have not yet been established. In contrast, human medicine has actively advanced BESS through numerous studies validating its safety and effectiveness [33,34,35]. Recent research in this field has revealed several advantages of BESS [15,36,37,38,39,40,41]. It has been shown to result in faster discharge times than microendoscopic spinal surgery and offers superior decompression of the spinal canal and a wider working space and length compared to microendoscopic spinal surgery and UESS [15,21,37,39,42,43,44,45]. Moreover, it requires a shorter operative time and is more familiar to surgeons than the UESS [44,45,46]. These benefits have been increasingly validated in contemporary medical research.

BESS involves two portals obliquely approaching the disc area, creating a precise triangular configuration [47]. This technique and its indications were based on anatomical standards [47,48]. Significant anatomical differences exist between humans and dogs in vertebral body size and shape, facet joint configuration, spinal canal shape, transverse process position, and angle and extent of the pedicles and lamina [49]. Back morphology also differs: broad and flat in humans, but more rounded in dogs, which complicates the direct adaptation of human techniques in veterinary surgery [48]. In addition, BESS requires specialized instruments beyond those used in the UESS. Collectively, these anatomical and technical differences highlight the need for a standardized methodological framework to safely and reproducibly adapt BESS in dogs.

Considering the current situation, this experimental study aimed to establish and validate a reproducible and standardized methodological approach for performing BESS in dogs rather than merely applying the technique. A modified artificial disc was used to create an IVDD model targeting the most commonly affected T12–13 intervertebral space. The experimental design followed the mini-hemilaminectomy osteotomy technique, prioritizing maximal preservation of the articular facet and minimizing bone removal to enhance spinal stability [50,51,52]. Subsequently, a specialized CT-based measurement method was developed to define the anatomical indications and portal geometries required for the BESS approach. Through this methodological standardization, this study sought to provide foundational data for adapting BESS to veterinary spinal surgery and to support its future clinical application as a minimally invasive technique in dogs.

## 2. Materials and Methods

### 2.1. Specimens

Thirteen medium-sized canine cadavers (mean weight: 10.67 ± 1.26 kg) were used in this study. Specimens with spinal diseases, vertebral malformations, or surgical history were excluded, and preoperative computed tomography (CT) was performed for further verification. The cadavers were categorized into a pilot group (*n* = 3; mean body weight: 10.60 ± 0.79 kg) and a main group (*n* = 10; mean body weight: 10.69 ± 1.37 kg). The pilot group was used to understand the learning curve, whereas the main group was used after the surgeons became familiar with the procedure. Both groups were subjected to identical surgical protocols with the exception of increased surgical experience. Group classification was based on a significant reduction in surgical time and subjective assessment of procedural familiarity.

### 2.2. Specimen Preparation

Because the canine cadavers used in this study did not exhibit IVDD, a disc extrusion model was established by injecting the fluorescently dyed artificial disc material with methylene blue (FDADM-MB) into the T12–13 intervertebral disc space. FDADM-MB consisted of distilled water, agarose (0.75 g; USB, Cleveland, OH, USA), barium sulfate (2 g; Daejung, Busan, Republic of Korea), and methylene blue (2 mL; Daejung, Busan, Republic of Korea). Its composition was modified from the protocols described by Lockwood and Moon to achieve a viscosity similar to that of native disc material and enable clear visualization using CT (Aquilion Lightning 80, Canon Medical Systems Corporation, Tochigi, Japan) and endoscopy (Endovision, Daegu, Republic of Korea) [9,12].

Each cadaver was positioned in ventrodorsal recumbency, and a midline incision was made from the xiphoid process to the last mammary gland. The ventral surface of the thoracic vertebrae was exposed after lateral retraction of the intestine and cranial retraction of the liver. The T13 vertebral body was identified by palpation of the thirteenth rib, and a needle was advanced cranially from the midpoint of T13 to access the T12–13 disc space. A total of 0.3 mL of FDADM-MB was injected to simulate disc extrusion, with the injection direction (left or right) randomized in all cases.

### 2.3. Surgical Instruments for BESS

After skin incision, BESS-specific instruments (Endovision, Daegu, Republic of Korea) were applied through the two portals. Muscle splitting of the accessory process was initiated with a dilator, followed by soft tissue dissection with a detacher (Endovision, Daegu, Republic of Korea) and retraction using both a standard and universal retractor (Endovision, Daegu, Republic of Korea). The scope portal included a 0°, 6.0 mm diameter, and 117.5 mm-long endoscope and sheath (Endovision, Daegu, Republic of Korea) for intraoperative visualization. Various instruments were used through the working portal, including forceps (Endovision, Daegu, Republic of Korea), punches (Endovision), and periosteal penetration tools (Richard Wolf GmbH, Knittlingen, Germany).

**Figure 1:**
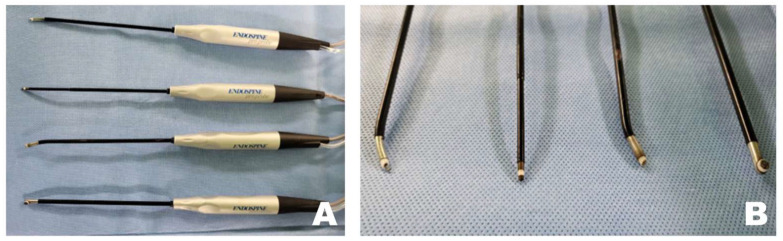
Specialized radiofrequency (RF) tools for biportal endoscopic spinal surgery. (**A**) RF tool that coagulates tissue using electromagnetic waves to maintain a clear surgical field. (**B**) Various shapes of the tip portion of the RF tool.

Tools that contribute significantly to safer and more time-efficient surgeries include radiofrequency (RF) tools (Endovision, Daegu, Republic of Korea), which coagulate tissues using electromagnetic waves to maintain the working space and clot bleeding areas (Figure 1). A burr (Medtronics, Minneapolis, Ireland), shaver (Arthrex, Naples, FL, USA), and microinstruments, including a bone awl and bone punch (Endovision, Daegu, Republic of Korea), were used to perform the osteotomy. Finally, safe disc removal was achieved using micro-rongeurs and microprobes (Rita, Mülheim, Germany). Irrigation pressure was maintained within a low and stable range of approximately 25–40 mmHg using a pressure-controlled pump throughout the procedure. This setting allowed consistent visualization and working space maintenance during endoscopic manipulation while avoiding excessive epidural pressure.

### 2.4. Surgical Procedure

**Figure 2:**
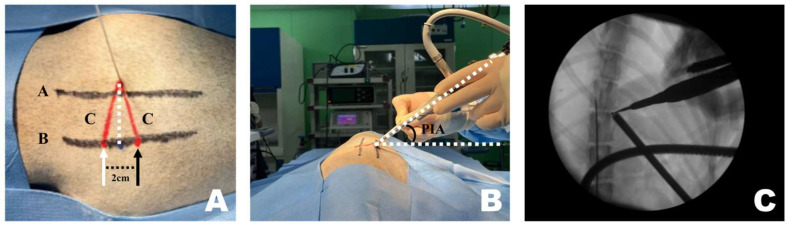
Application of the CT-based measurement diagram to the actual patient. (**A**) Line A represents the lateral margin connecting the spinous processes, similar to the diagram. Since the pin was positioned at the center of the spinous process and line A connected the lateral margins, line A was drawn more laterally. The dotted line represents the portal insertion distance for drawing a line. The white arrow indicates the starting point of the scope portal, and the black arrow indicates the starting point of the working portal. Consistent with the diagram, the red line represents line C, corresponding to the portal insertion pathway. (**B**) The required inclination of the scope and working instrument is maintained at the portal insertion angle while being inserted along line C. (**C**) Verification using the dorsoventral view imaging confirmed that the scope and working instrument were correctly inserted into the accessory process and that an accurate triangle was formed. PIA, Portal Insertion Angle.

CT-based measurement values were obtained during surgical procedures. A pin was placed in the T12–13 intervertebral space. Line A, a straight line connecting the lateral margins of the spinous process in the surgeon’s direction, was established. Subsequently, parallel to line A, line B was drawn from the PID pin. At the PID on line B, two marks were made 1 cm apart, serving as insertion points for the scope and working portals, which were 2 cm apart [47,53,54]. Finally, line C was drawn from each portal to the pin, forming an isosceles triangular PIP (Figure 2A).

A 1 cm skin incision was made at each of the two portal insertion points, spaced 2 cm apart. First, using the working portal, a dilator was inserted along line C to identify the accessory process and split the surrounding muscles. A detector was used to dissect muscles and ligaments attached to the accessory process. The same procedure was performed for the scope portal. While separating the muscles, considerable force was applied downward, following the initially planned PIP. However, while identifying the accessory process and separating the muscles, the PIP appeared parallel to the spinous process.

Once sufficient space was created, the endoscope and instruments were inserted along line C and angled according to the PIA (Figure 2B). Correct placement was verified using fluoroscopy (Figure 2C). The pin was removed or left in place after confirming accurate placement using fluoroscopy.

**Figure 3:**
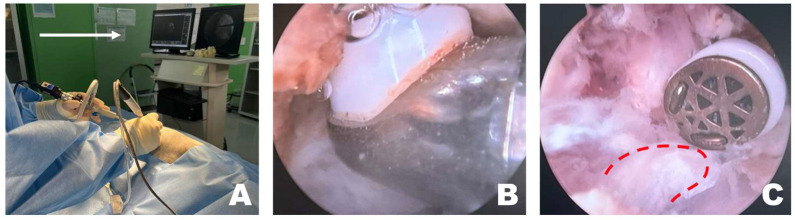
Removal of soft tissue in the endoscopic view toward the left. (**A**) The surgeon performed the surgery while facing the monitor in front. The white arrow indicates the direction of the surgeon’s gaze during the surgery. (**B**) When radiofrequency (RF) tools were applied to the epaxial muscles in the left endoscopic view, tissue cauterization, vessel coagulation, and bubble formation were observed. (**C**) RF tools were used to dissect soft tissue attached to the accessory process, which became visible (red dotted line) after separation of the longissimus thoracis muscle.

After verifying correct placement, the surgeon focused on the monitor connected to the scope (Figure 3A). If the working space was clearly visible through the scope, forceps and punches were used to remove the epaxial muscles attached to the accessory process or any surrounding tissue that hindered visualization. Following the excision of the larger epaxial muscles, smaller tissues that were difficult to remove with forceps and punches were cleared using RF tools suitable for the situation, creating a cleaner view of the working space (Figure 3B). The longissimus thoracis was incised using an appropriate RF tool to expose the accessory process (Figure 3C).

**Figure 4:**
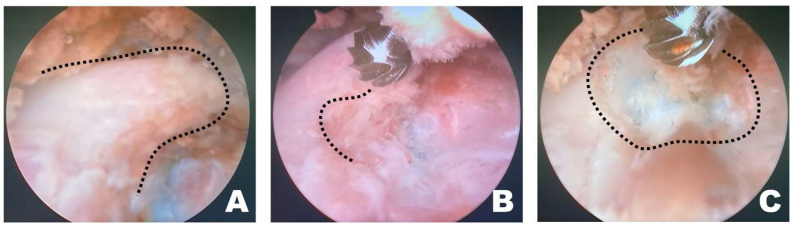
Osteotomy of the accessory process in the endoscopic view toward the left. (**A**) The dashed line indicates the T12 accessory process. Clear and distinct observation was possible; the fluorescently dyed artificial disc material with methylene blue was observed at this time. (**B**) The dashed line marked the initiation point of osteotomy. Osteotomy was performed by securely placing the burr on the widest surface of the caudal aspect of the accessory process. (**C**) The dashed line indicates the surgical window. Additional osteotomy was extended along the dashed line.

Once a clear view of the working space for accessory process osteotomy was established (Figure 4A), burr osteotomy was performed to ensure that it was securely seated on the wider surface of the caudal aspect of the accessory process to prevent slipping (Figure 4B). Thus, the osteotomy site served as the surgical window (Figure 4C). If nerve exposure and potential nerve damage increased owing to insufficient burr osteotomy, an additional osteotomy was performed with a shaver. If precise soft tissue trimming was performed, FDADM-MB could be observed through the transforaminal site without osteotomy of the accessory process, alongside the epidural fat (Figure 4).

**Figure 5:**
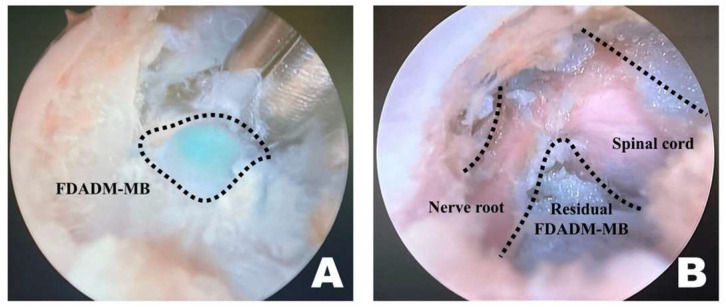
Identification and removal of the fluorescently dyed artificial disc material with methylene blue (FDADM-MB) in the endoscopic view toward the left. (**A**) The FDADM-MB was clearly visible in blue through the completed surgical window after removing various intervertebral foraminal and intraforaminal ligaments using forceps. (**B**) Repeated use of the micro-ronguer to remove all removable disc material. A clear observation of the spinal cord and nerve root was achieved, and the residual FDADM-MB remained after removal.

Upon overserving the FDADM-MB, after removing intervertebral foraminal and intraforaminal ligaments, it was removed using a microngeur (Rita, Mulheim, Germany) (Figure 5) [55]. FDADM-MB on the ventral aspect of the spinal cord, which could not be removed with the microngeur, was extracted using a microprobe (Rita, Mulheim, Germany). After maximal removal, the probe was inserted under the spinal cord, slightly retracted dorsally, and checked for the remaining FDADM-MBs.

Several technical observations related to portal placement, body conformation, and instrument handling were recorded during the surgical procedure.

**Figure 6:**
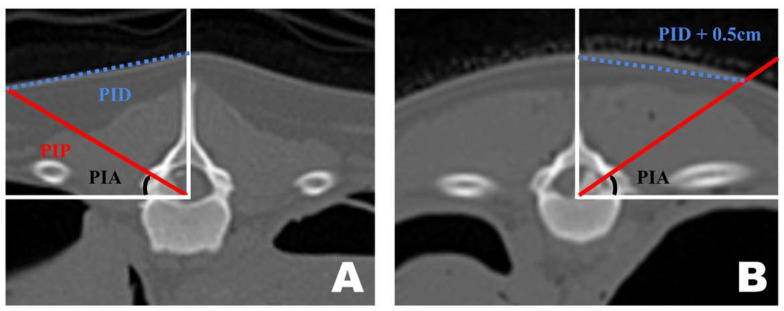
Differences in portal insertion distance (PID) measurement values in dogs with different body types as seen in the frontal view of a transverse computed tomography scan. (**A**) PID measurement in a thin-bodied dog. (**B**) PID measurement in a thick-bodied dog. Since the actual distance drawn on the skin differed during surgery, an additional 0.5 cm was added to the premeasured PID value for portal insertion.

Preoperative CT-based PID measurements are generally reliable for guiding portal insertion. However, variations in body shape introduced discrepancies between the premeasured PID and the actual lateral distance on the skin surface during surgery (Figure 6). In dogs with shorter torsos and thicker paraspinal tissues, the premeasured PID did not fully reflect the true lateral distance required for portal insertion. By contrast, in thinner dogs with flatter dorsal contours, the CT-based PID values corresponded more closely to the actual skin distance. In cases with a curved dorsal surface, an additional lateral adjustment of approximately 0.5 cm was applied to the premeasured PID to achieve accurate portal placement.

**Figure 7:**
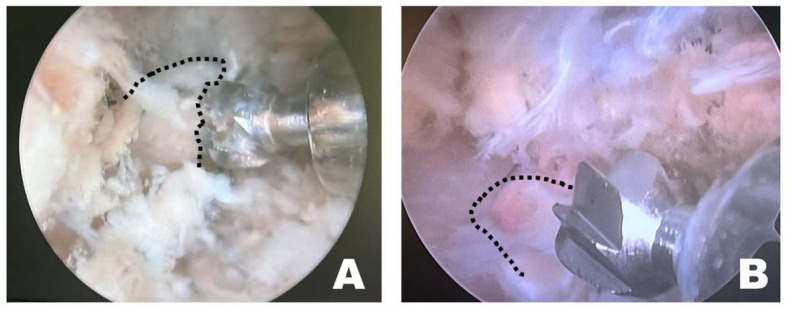
Endoscopic observation, depending on the side of the surgical intervention site. (**A**) On the patient’s left side, the osteotomy was initiated by anchoring at the wider caudal portion of the accessory process. (**B**) On the patient’s right side, the osteotomy was initiated from the middle of the accessory process without secure anchoring. The dashed line indicates the accessory process.

Instrument handling during osteotomy differed depending on the surgical approach (Figure 7). When the procedure was performed on the left side of the dog, the osteotomy was initiated by anchoring the burr to the wider caudal portion of the accessory process, which allowed stable initial anchorage during progressive bone removal. In contrast, when the procedure was performed from the right side, the initial anchorage was less stable, and osteotomy was more frequently initiated from a more central portion of the accessory process. All procedures were performed with the dogs maintained in the dorsoventral position by a right-handed surgeon.

**Figure 8:**
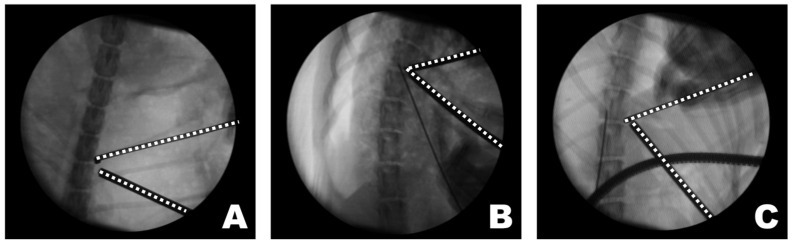
Observations of the triangular formation of the endoscope and working instrument from the dorsoventral view of the fluoroscopy. (**A**) In an open triangle formation, a working space was not created, and the instruments were not visible. (**B**) In a perfect triangle formation, the working space was appropriately created at the target point, and the visualization of the instrument’s tip was accurate. (**C**) In a close triangle formation, while a working space was formed, it was not at the target point, and the shaft of the instrument, rather than its tip, was observed. The dashed lines indicate the tips of the endoscope and the working instrument.

The triangular portal arrangement influenced working space formation and endoscopic visualization (Figure 8). When an appropriate triangular configuration was achieved, a working space was created at the target point, and the tip of the instrument remained consistently visible. In this configuration, instrument manipulation and visualization could be maintained simultaneously within the endoscopic field. In the open triangular configuration, a working space was not formed, and the instruments were not visible. This configuration was associated with failure to establish a functional working corridor at the target point. Although the working space was formed in a closed triangular configuration, it was located away from the target point, and only the shaft of the instrument, rather than the tip, was observed. As a result, precise manipulation at the accessory process was limited despite the presence of a working space.

### 2.5. Data Collection

#### 2.5.1. Preoperative CT-Based Measurement

The objective of this study was to perform a successful and appropriate mini-hemilaminectomy targeting the accessory process. Two preoperative CT-based measurements were performed to define accurate portal alignment with the accessory process. These measurements included the portal insertion distance (PID), which determines the distance from the spinous process to the starting point of the portal insertion and guides the appropriate skin incision site, and the portal insertion angle (PIA), which represents the angle required to approach the accessory process from the skin incision site. These measurements were obtained using preoperative CT in the dorsoventral (DV) position. A 160-channel multidetector row CT scanner (Canon, Tokyo, Japan) was used, shooting at 110 kV and 36 mAs with 0.5 mm thick slices. Statistical analysis was conducted using SPSS version 29.0.1.0 (SPSS Inc., Chicago, IL, USA). Comparisons were performed using the Mann–Whitney test, and statistical significance was set at *p* < 0.05. Furthermore, three-dimensional (3D) reconstruction of the bone and FDADM-MB was implemented using 3D Slicer version 5.5.0.

#### 2.5.2. Perioperative Evaluation

To measure the surgical time, the procedure was categorized into five phases based on the steps that involved significant changes in the use and manipulation of instruments through the working portal. These phases were measured sequentially as portal complete time (PCT), accessory exposure time (AET), osteotomy completion time (OCT), disc removal time (DRT), and total surgical time (TST). An assistant other than the designated surgical assistant manually recorded the time at the end of each step. The technical difficulty of the BESS technique, based on preoperative CT-based measurements, was subjectively described and recorded after each surgery. Before the subsequent surgery, technical notes were reviewed as reminders.

#### 2.5.3. Postoperative Measurement

For postoperative evaluation, CT scans in the DV position were 3D reconstructed to visualize the bone after surgery. This process facilitated measurement of the surgical window, injected FDADM-MB volume, residual FDADM-MB volume, and residual FDADM-MB percentage. A 160-channel multidetector row CT scanner (Canon, Tokyo, Japan) was used, shooting at 110 kV and 36 mAs with 0.5 mm thick slices. Statistical analysis was conducted using SPSS version 29.0.1.0 (SPSS Inc., Chicago, IL, USA). Comparisons were performed using the Mann–Whitney test, and statistical significance was set at *p* < 0.05. Additionally, 3D reconstruction of the bone and FDADM-MB were performed using 3D Slicer version 5.5.0.

### 2.6. Graphical Abstract

We ChatGPT (OpenAI, GPT-4–based model) was used to assist in the preparation of the graphical abstract. The tool was used solely for generating graphical abstract. The authors reviewed, edited, and finalized the graphical abstract and take full responsibility for the content.

## 3. Results

### 3.1. Surgical Plan Using CT-Based Measurements

**Figure 9:**
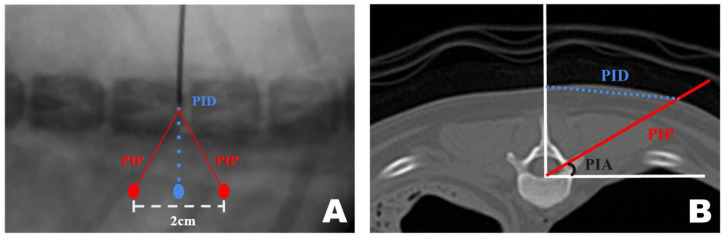
Computed tomography (CT)-based measurement method applied for precise mini-hemilaminectomy. (**A**) Transverse CT view of the T12 accessory process. (**B**) Diagram of approach indicating the dorsoventral view of the T12-13 vertebra bone as seen during actual surgery through the fluoroscopy. PID, Portal Insertion Distance; PIP, Portal Insertion Pathway; PIA, Portal Insertion Angle.

A transverse view of the T12 accessory process was obtained on the CT scan. A line was drawn from the midpoint of the ventral portion of the spinal canal to the T12 spinous process. Subsequently, a perpendicular line was drawn on the floor. A line connecting the accessory processes was drawn at the intersection of the two lines, which represented the actual portal insertion pathway (PIP). At this point, the distance from the skin portion on the spinous process line to the PIP was measured, which became the PID, determining the actual site of the skin incision. Furthermore, the angle between the floor line and PIP, which was maintained while inserting the portal from the PID-defined skin incision site to the accessory process, became the PIA (Figure 9A).

The approach indication diagram was based on the DV position when the surgeon uses a CT-based measurement method during surgery. The distance between the two portals was 2 cm, and the procedure was conducted while maintaining a triangular formation as the portals were positioned (Figure 9B).

### 3.2. Surgical Site Evaluations

**Figure 10:**
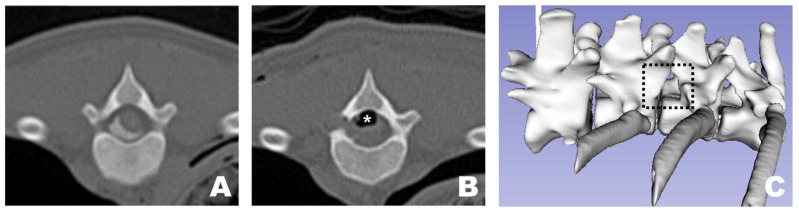
Osteotomy site as observed in CT and 3D reconstruction view at the transverse view of a CT scan. (**A**) Pre-operative appearance with the FDADM-MB properly implemented. (**B**) Post-operative view showing appropriate osteotomy of the accessory process and removal of the FDADM-MB. Due to the nature of the cadaver, the air bubbles (white asterisk) formed above the spinal cord did not resolve after surgery. (**C**) The black dotted square highlights the area in the 3D reconstruction view where the accessory process is completely removed, clearly revealing the lateral aspect of the spinal cord.

In all dogs, FDADM-MB was clearly visualized through the endoscopic view without requiring any additional incisions beyond the initial 1 cm skin incision at each portal. Following the completion of the preparatory steps, the scope was stabilized with the left hand, and disc removal was performed via the working portal. Osteotomy of the accessory process was successfully performed in all patients, as confirmed by postoperative CT imaging and 3D reconstruction (Figure 10). In two of the 13 dogs, additional osteotomy was required beyond the accessory process: the articular process (Dog 1) and vertebral body (Dog 3).

Regardless of the direction of implementation of the IVDD model, a clear visualization of the spinal cord and nerve roots was achieved in all subjects.

### 3.3. Preoperative CT-Based Measurement Assessments

Measurements were successfully performed in all 13 dogs (Table 1). Using the CT-based measurement method, the mean PIA was 31.00 ± 2.79°, and the mean PID was 32.95 ± 3.05°.

The mean PIA in the pilot and main groups was 31.05 ± 1.89° and 30.99 ± 3.00°, respectively, with no significant difference (*p* = 0.811). The mean PID in the pilot and main groups was 33.46 ± 3.36 mm and 32.79 ± 2.93 mm, respectively, also showing no significant difference (*p* = 0.937).

### 3.4. Postoperative CT Assessments

In all 13 dogs, successful 3D reconstruction was not only applied but also resulted in measurable values being evaluated (Table 2). The average injected FDADM-MB volume measured was 169.26 ± 30.06 mm^3^, and the average residual FDADM-MB volume was 11.39 ± 4.64 mm^3^. Considering the residual FDADM-MB volume as the remaining disc volume after removal, the average residual FDADM-MB percentage was calculated to be 6.89 ± 1.66%. The average surgical window created for disc removal through osteotomy was 33.06 ± 13.90 mm^2^.

In the pilot group, the average injected FDADM-MB volume was 166.25 ± 3.67 mm^3^, the average residual FDADM-MB volume was 12.84 ± 2.10 mm^3^, and the average residual FDADM-MB percentage was 7.71 ± 1.19%. The average surgical window was 42.30 ± 17.81 mm^2^. In the main group, the average injected FDADM-MB volume was 170.17 ± 34.16 mm^3^, the average residual FDADM-MB volume was 10.95 ± 1.97 mm^3^, and the average residual FDADM-MB percentage was 6.65 ± 1.70%. The average surgical window was 30.06 ± 13.90 mm^2^. No significant differences were observed in the injected FDADM-MB volume, residual FDADM-MB volume, residual FDADM-MB percentage, or surgical window between the pilot and main groups.

### 3.5. Postoperative Surgical Outcomes

Successful surgical time recordings were performed for all 13 dogs. The surgical procedure was categorized into five stages, and the assistant responsible for recording the time recorded the duration at which the surgeon indicated the completion of each stage. Two methods were used to measure time: cumulative (adding the time of each stage to that of the previous stages) and individual surgical time (recording the duration of each stage independently).

Across all dogs, the mean PCT, AET, OCT, DRT, and TST were 15.97 ± 15.16 min, 25.26 ± 20.55 min, 47.92 ± 25.93 min, 57.65 ± 26.69 min, and 62.05 ± 26.74 min, respectively.

In the pilot group, the mean PCT, AET, OCT, DRT, and TST were 42.64 ± 7.01 min, 61.21 ± 6.46 min, 92.48 ± 12.64 min, 104.23 ± 8.01 min, and 107.75 ± 8.79 min, respectively. In the main group, the mean PCT, AET, OCT, DRT, and TST were 7.96 ± 2.50 min, 14.47 ± 5.67 min, 34.552 ± 7.16 min, 43.67 ± 7.78 min, and 48.34 ± 9.60 min, respectively.

**Figure 11:**
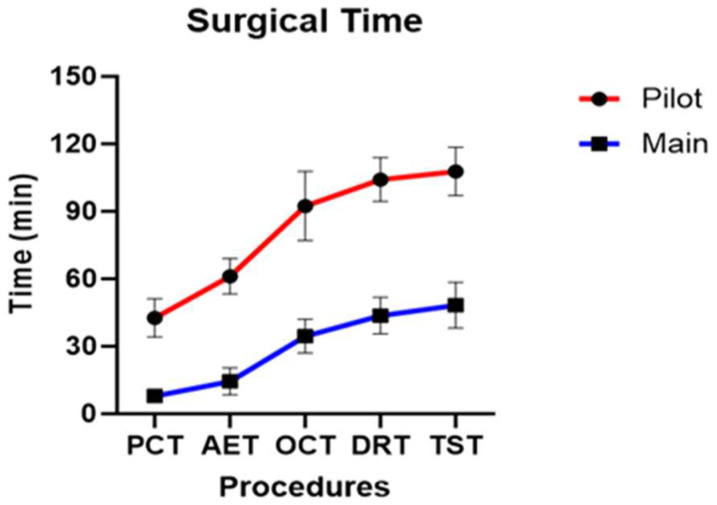
Profile graph of cumulative surgical time calculated for the five stages of surgery.

Statistically significant differences were observed between the pilot and main groups for all five stages of cumulative surgical time (*p* < 0.001) (Figure 11). When individual stage times were analyzed, statistically significant differences were observed in PCT (*p* = 0.007), AET (*p* = 0.049), while OCT (*p* = 0.469), DRT (*p* = 0.937) and TST (*p* = 0.692) did not show a statistically significant difference. The stages that showed statistically significant differences in both cumulative and individual surgical times were PCT and AET.

## 4. Discussion

This study serves as an extension of a previous investigation on the application of the BESS technique in veterinary medicine, in which mini-hemilaminectomy of the accessory process was successfully performed in all subjects. The accessory osteotomy site was clearly visualized endoscopically using a 0° scope. In human medicine, several studies have reported that a 30° scope provides superior visualization during endoscopic spinal procedures [39,56,57]. A comparative evaluation of a 30° scope in veterinary BESS, particularly by surgeons accustomed to 0° endoscopy, may facilitate further investigation into the optimization of the osteotomy site and surgical window formation in the endoscopic view. In the present cadaveric model, a 0° endoscope provided sufficient visualization of the accessory process, osteotomy site, and FDADM-MB through a transforaminal approach, allowing accurate surgical window formation and disc material removal. The relatively shallow operative depth and direct line-of-sight toward the accessory process in medium-sized dogs likely contributed to the adequacy of a 0° viewing angle.

Based on the FDADM-MB removal measurements, surgical window size, and duration of surgery, the BESS technique demonstrated feasibility as an alternative MISS technique in veterinary medicine. In addition, FDADM-MB was successfully visualized using both CT and endoscopy. The injected FDADM-MB volume closely matched the reported extruded disc volumes in medium-sized canine patients with IVDD, indicating successful establishment of an IVDD model using FDADM-MB.

To the best of our knowledge, this is the first study to successfully implement and visualize FDADM-MB within the spinal canal using endoscopy. Previous studies by Lockwood and Moon used barium-containing artificial discs to enable the CT-based visualization of disc materials [9,12]. In this study, the injected FDADM-MB volume was within the reported range of extruded disc volumes in medium-sized canine IVDD cases (60.9–240 mm^3^; median, 120 mm^3^) [58], further supporting the validity of the experimental IVDD model.

Although the IVDD model was successfully established, adapting BESS to veterinary medicine involved several technical challenges related to its first-time application and anatomical differences that limit direct translation of human surgical techniques [19,34,56,59,60,61]. While the isosceles triangular portal configuration, with the two portals positioned approximately 2 cm apart, was adapted from human BESS practice [47,53,54], most other procedural parameters required modification and optimization specific to canine anatomy.

Minor variation in PID related to body conformation was observed; however, a simple lateral adjustment of approximately 0.5 cm to the pre-measured PID was sufficient and did not result in any detectable difference in surgical feasibility or outcomes. This finding supports the practical reliability of the CT-based measurement method for BESS portal planning in medium-sized dogs.

Surgical ergonomics associated with BESS may also be influenced by surgeon-related factors such as hand dominance [39,59]. In this study, all procedures were performed in the dorsoventral position by a right-handed surgeon, and qualitative intraoperative observations suggested that the stability of instrument anchorage during osteotomy varied depending on the side of the surgical approach. Although this study did not quantitatively assess whether the side of surgical intervention influenced perioperative processes or outcomes, the potential interaction between surgeon hand dominance and the approach side warrants further investigation to optimize procedural efficiency and ergonomics.

Maintaining an accurate triangular portal configuration is a fundamental technical requirement of BESS to preserve a stable endoscopic field and efficient irrigation flow [47,62]. In BESS, irrigation fluid introduced through the scope portal drains naturally toward the working portal without suction, making precise triangular alignment critical for maintaining a clear and directional fluid flow. Inadequate portal convergence may compromise visualization and working space formation, potentially prolonging the surgical time and increasing soft tissue manipulation [47,63,64]. Specifically, an open triangular configuration may prevent the formation of an effective working space at the target point, whereas premature convergence of the portals can result in a working space formed away from the accessory process, limiting visualization of the instrument tip. These technical considerations underscore the importance of precise portal planning and intraoperative spatial awareness in veterinary BESS.

Previous reports of open mini-hemilaminectomies in medium-sized dogs demonstrated a residual disc volume of approximately 7.7% [58]. In this study, small amounts of FDADM-MB remained in all the groups following decompression. Compared with UESS, which required a mean surgical window area of 58.95 ± 18.13 mm^2^ and a mean surgical time of 58.00 ± 18.06 min using a similar IVDD model, BESS achieved effective decompression with a smaller osteotomy area [9,34,56,59,60,61]. Although the overall mean surgical time in both groups was longer than that reported in previous UESS studies, the main group, which surpassed the learning curve, had shorter operative times. This suggests that experienced surgeons may perform BESS more efficiently while minimizing bone removal, potentially reducing anesthesia duration, and preserving native spinal structures. Notably, previous studies did not include separate analyses of the pilot and main groups.

To our knowledge, this study is the first in veterinary medicine to compare the learning curves of a pilot and main groups within the context of MISS. A significant reduction in surgical time was observed beginning with the fourth dog, coinciding with improved familiarity with the endoscopic instruments and enhanced spatial orientation. The additional osteotomies required in early cases were not observed in subsequent procedures, suggesting improved technical precision. Among the five defined surgical stages, significant reductions were particularly evident in PCT and AET, both of which involve challenges related to portal placement, endoscope control, and accurate exposure of the accessory process. These findings indicated that the early phase of the procedure is the most technically demanding part of the learning curve.

Notably, no significant differences were observed between the pilot and main groups in PIA or PID, supporting the reliability of the CT-based measurement method, even during the early learning phase. Although the surgical window size did not differ significantly between groups, the main group demonstrated a tendency toward smaller osteotomy areas. Collectively, these findings suggest that veterinary BESS has a relatively short learning curve, with technical proficiency markedly improving after several procedures.

Although the present study was conducted using cadaveric specimens, the findings allow consideration of potential postoperative management implications when BESS is applied in live canine patients. Compared with conventional open approaches, BESS involves reduced muscle dissection, a smaller osteotomy, and preservation of surrounding osseous and soft tissue structures, which may translate into reduced postoperative pain, decreased soft tissue trauma, and earlier functional recovery. In addition, preservation of the articular facet and a minimized surgical window may contribute to maintaining postoperative spinal stability, particularly in thoracic spinal surgery. While these clinical outcomes cannot be directly assessed in a cadaveric model, discussing these potential postoperative implications provides important clinical context and underscores the need for future in vivo studies.

This study had some limitations. First, all procedures were performed on cadavers, and tissue characteristics may differ from those of live animals, potentially influencing the intraoperative handling and interpretation of outcomes. In particular, the cadaveric model inherently excludes key intraoperative challenges encountered in live biportal endoscopic spinal surgery, most notably active epidural venous plexus bleeding. In clinical settings, such bleeding can rapidly obscure the endoscopic visual field and requires continuous hemostasis and dynamic irrigation management, factors that cannot be reproduced in cadaveric specimens. These real-world conditions may substantially affect visualization quality, working space maintenance, and procedural workflow during live surgery. Second, the IVDD model was applied to morphologically normal canine spines, and the results may not be generalizable to patients with vertebral malformations or those who have undergone previous spinal surgery. Third, postoperative evaluation relied solely on CT imaging; without magnetic resonance imaging (MRI), potential spinal cord injury could not be assessed. Although CT provides excellent visualization of osseous anatomy and is sufficient for preoperative planning of bony landmarks and portal trajectories, it has inherent limitations in evaluating soft tissue structures. Both preoperative and postoperative CT-based assessments are insufficient to detect subtle spinal cord pathology, including iatrogenic micro-trauma or thermal injury, and the absence of histopathological evaluation further limits microscopic assessment of neural tissue response. Therefore, reliance on CT alone may restrict comprehensive neurological evaluation in this study. Fourth, only a 0° endoscope was used; comparative studies using a 30° scope are warranted. Fifth, although conceptual comparisons were made with other MISS techniques, such as microscopic surgery and UESS, no direct statistical comparisons were performed. Finally, all procedures were conducted by a single surgeon experienced in open surgery and cadaveric UESS, but without prior training in laparoscopy or arthroscopy, suggesting that previous endoscopic experience may further shorten the learning curve when adopting BESS.

## 5. Conclusions

This study established a standardized surgical methodology for applying BESS in veterinary medicine. Furthermore, we successfully created an IVDD model by injecting FDADM-MB into the T12–13 intervertebral space, which was clearly visualized in both CT and endoscopic views. This experimental model enabled consistent application of the BESS technique for mini-hemilaminectomy and disc material removal using a CT-based measurement approach. Moreover, the findings suggest that given the relatively short learning curve, BESS can be performed more efficiently as surgical familiarity increases. In addition, as a practical clinical guideline, increasing the PID by approximately 0.5 cm in dogs with thicker paraspinal tissues may serve as a useful rule of thumb to maintain adequate visualization during the procedure.

Despite these limitations, this study represents an important step toward the future application of BESS in other spinal disc models and live animal subjects.

## Figures and Tables

**Table 1:** PIA and PID values measured through the CT-based measurement method.

	PIA (°)	PID (mm)
Pilot study	31.05 ± 1.89	33.46 ± 3.36
Main study	30.99 ± 3.00	32.79 ± 2.93
*p*-value	0.811	0.937
All	31.00 ± 2.79	32.95 ± 3.05

Data are presented as mean ± SD. PIA, portal insertion angle; PID, portal insertion distance; CT, computed tomography; SD, standard deviation.

**Table 2:** Values related to the FDADM-MB and surgical window from osteotomy, as measured through 3D reconstruction from CT imaging.

	Injected FDADM-MB Volume (mm^3^)	Residual FDADM-MB Volume (mm^3^)	Residual FDADM-MB Percentage (%)	Surgical Window (mm^2^)
Pilot study	166.25 ± 3.67	12.84 ± 2.10	7.71 ± 1.19	42.30 ± 17.81
Main study	170.17 ± 34.16	10.95 ± 1.97	6.65 ± 1.70	30.54 ± 11.96
*p*-value	1.000	0.238	0.217	0.370
All	169.26 ± 30.06	11.39 ± 4.64	6.89 ± 1.66	33.06 ± 13.90

Data are presented as mean ± SD. FDADM-MB, fluorescently dyed artificial disc material with methylene blue; CT, computed tomography; SD, standard deviation; 3D, three-dimensional.

## Data Availability

Data available on request due to restrictions, e.g., privacy or ethical.

## Abbreviations

AET	Accessory Exposure Time
BESS	Biportal Endoscopic Spinal Surgery
CT	Computed Tomography
DRT	Disc Removal Time
DV	Dorsoventral
EDA	Electrodermal Activity
FDADM-MB	Fluorescently Dyed Artificial Disc Material with Methylene Blue
HDSS	Hyperhidrosis Disease Severity Scale
IVDD	Intervertebral Disc Disease
MISS	Minimally Invasive Spinal Surgery
OCT	Osteotomy Completion Time
PCT	Portal Completion Time
PIA	Portal Insertion Angle
PID	Portal Insertion Distance
PIP	Portal Insertion Pathway
RF	Radiofrequency
TST	Total Surgical Time
UESS	Uniportal Endoscopic Spinal Surgery
